# ADAM10 sheddase activation is controlled by cell membrane asymmetry

**DOI:** 10.1093/jmcb/mjz008

**Published:** 2019-02-12

**Authors:** Florian Bleibaum, Anselm Sommer, Martin Veit, Björn Rabe, Jörg Andrä, Karl Kunzelmann, Christian Nehls, Wilmar Correa, Thomas Gutsmann, Joachim Grötzinger, Sucharit Bhakdi, Karina Reiss

**Affiliations:** 1 Department of Dermatology, University of Kiel, Kiel, Germany; 2 Institute of Biochemistry, University of Kiel, Olshausenstraße 40, Kiel, Germany; 3 Hamburg University of Applied Science, Ulmenliet 20, Hamburg, Germany; 4 Physiological Institute, University of Regensburg, Universitätsstraße 31, Regensburg, Germany; 5 Forschungszentrum Borstel, Leibniz-Zentrum für Medizin und Biowissenschaften, Parkallee 10, Borstel, Germany

**Keywords:** ADAM10, activation, shedding, Anoctamin-6, phosphatidylserine, cell membrane asymmetry

## Abstract

Dysregulation of the disintegrin-metalloproteinase ADAM10 may contribute to the development of diseases including tumorigenesis and Alzheimer’s disease. The mechanisms underlying ADAM10 sheddase activation are incompletely understood. Here, we show that transient exposure of the negatively charged phospholipid phosphatidylserine (PS) is necessarily required. The soluble PS headgroup was found to act as competitive inhibitor of substrate cleavage. Overexpression of the Ca^2+^-dependent phospholipid scramblase Anoctamin-6 (ANO6) led to increased PS externalization and substrate release. Transfection with a constitutively active form of ANO6 resulted in maximum sheddase activity in the absence of any stimulus. Calcium-dependent ADAM10 activation could not be induced in lymphocytes of patients with Scott syndrome harbouring a missense mutation in ANO6. A putative PS-binding motif was identified in the conserved stalk region. Replacement of this motif resulted in strong reduction of sheddase activity. In conjunction with the recently described 3D structure of the ADAM10 extracellular domain, a model is advanced to explain how surface-exposed PS triggers ADAM10 sheddase function.

## Introduction

Disintegrin-like metalloproteases (ADAMs) control many important cellular functions through the release of membrane-anchored molecules from the cell surface (Reiss and Saftig, 2009; Saftig and Reiss, 2011). ADAM10 and ADAM17 are the best characterized members of the family. Deletion of the ADAM10 gene leads to embryonic lethality in mice due to disturbed Notch signalling (Hartmann et al., 2002). Several substrates have been identified implicating roles for the protease in a variety of pathophysiological processes. ADAM10 is the major sheddase of cell adhesion molecules including neuronal (N)-cadherin (Reiss et al., 2005), epithelial (E)-cadherin (Maretzky et al., 2005), and vascular-endothelial (VE)-cadherin (Schulz et al., 2008), but also releases the EGFR ligands betacellulin (BTC) and EGF (Sahin et al., 2004) and the low affinity IgE receptor CD23 (Weskamp et al., 2006; Lemieux et al., 2007; Le Gall et al., 2009; Pupovac et al., 2015). Moreover, the protease mediates the non-amyloidogenic α-secretase cleavage of the Alzheimer’s precursor protein. Dysregulated ADAM10 activity is assumed to play a central role in diverse pathologies including Alzheimer’s disease, allergic responses, and cancer development (Weskamp et al., 2006; Murphy, 2008; Marcello et al., 2017). This underlines the importance to understand how ADAM10 shedding activity is regulated. The constitutive release of ADAM10 substrates can be rapidly enhanced by cytosolic Ca^2+^ elevation, as elicitable by treatment of cells with Ca^2+^-ionophores, purinergic receptor agonists, or membrane-perturbating agents (Reiss and Bhakdi, 2012; Maretzky et al., 2015; Pupovac and Sluyter, 2016). How this information is relayed from the cell to the externally oriented proteinase has long remained an enigma, particularly because its cytoplasmic domain is dispensable for sheddase activation (Maretzky et al., 2015). Recent findings have led us to propose that the membrane itself regulates the sheddase function of the closely related ADAM17. In quiescent cells, phosphatidylcholine (PC) and sphingomyelin (SM) are present predominantly in the outer leaflet while the anionic phosphatidylserine (PS) is located almost exclusively in the inner leaflet, where it serves as essential co-factor for a number of membrane-bound enzymes such c-Src, MARCKS, and protein kinase C (Mulgrew-Nesbitt et al., 2006; Fadeel and Xue, 2009). Activation of scramblases, which mediate the randomized bidirectional transport of phospholipids, results in PS exposure. Two scramblases have recently been characterized: Ca^2+^-activated transmembrane protein 16 F (TMEM16F, also known as Anoctamin-6) (Suzuki et al., 2010) and Xkr8 (Suzuki et al., 2013), a caspase-sensitive protein activated in response to apoptotic stimuli (Bevers and Williamson, 2016).

We previously observed that classical triggers of ADAM17 induced transient breakdown of cell membrane asymmetry. Evidence for a two-step model of ADAM17 sheddase activation was obtained (Sommer et al., 2016a, b). Externalized PS interacts electrostatically with the membrane-proximal domain (MPD) of ADAM17. In a further step, the evolutionarily conserved stalk region of ADAM17, also denoted CANDIS (conserved ADAM seventeen dynamic interaction sequence) (Dusterhoft et al., 2015), associates via hydrophobic interaction with the membrane. These events, acting in concert, lead to substrate cleavage at the sites of membrane reorganization.

The question followed whether similar membrane-related events might also control ADAM10 function. In this communication, we report the results of experiments conducted with gain- and loss-of-function mutants of Anoctamin-6 and with rabbit erythrocytes that naturally express ADAM10 on their surface. Both models generated data that also indicated an important role for surface-exposed PS in regulating substrate cleavage by ADAM10. A triplet cluster of cationic amino acid residues had previously been identified as the PS-binding motif in the MPD of ADAM17 (Sommer et al., 2016b). A similar motif is present in the stalk region of ADAM10, which was also previously observed to bind to PS liposomes (Dusterhoft et al., 2015). Replacement of these positively charged residues led to marked reduction of sheddase function despite the presence of the mature ADAM10 mutant at the cell surface. The collective data indicate that the biological function of ADAM10 is centrally controlled by surface-exposed PS.

## Results

### Soluble phosphorylserine suppresses ADAM10 sheddase function

**Figure 1:**
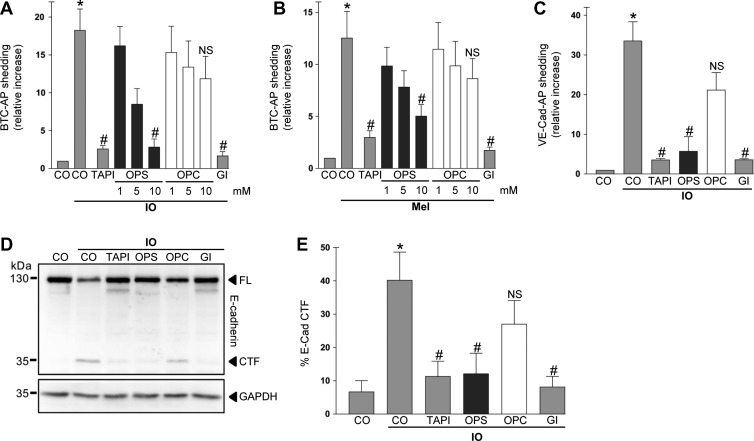
PS interaction is required for ADAM10 activation. (**A** and **B**) COS7 cells were transfected with the AP-tagged ADAM10 substrate BTC and stimulated with ionomycin (IO, 1 μM; **A**) or melittin (Mel, 0.5 μM; **B**) for 30 min. Shedding was dose-dependently reduced by addition of the competing phosphatidylserine head group (OPS) but not by the head group of phosphatidylcholine (OPC). OPS (10 mM), broadspectrum metalloprotease inhibitor TAPI-1 (10 μM), and ADAM10 inhibitor GI (3 μM) significantly abrogated the induced shedding. (**C**) COS7 cells were transfected with the AP-tagged ADAM10 substrate VE-cadherin and stimulated with ionomycin for 30 min in the presence of OPS (10 mM), OPC (10 mM), TAPI-1 (10 μM), or ADAM10 inhibitor GI (3 μM) and analysed for substrate shedding. (**D**) Shedding of full-length (FL) E-cadherin was monitored by immunoblot analysis. HaCaT keratinocytes were stimulated with ionomycin (1 μM) in the presence of TAPI-1 (10 μM), OPS (10 mM), OPC (10 mM), or ADAM10 inhibitor GI (3 μM). (**E**) Densitometric quantification of E-cadherin C-terminal fragment (CTF) generation of three independent western blots. * indicates a significant increase compared to unstimulated cells; # indicates significant decrease compared to stimulated control (*P* < 0.05, *n* = 3; ±SEM). NS, no significant difference. Data were analysed by one-way analysis of variance and Bonferroni multiple comparison *post hoc* test. CO, control.

To assess whether ADAM10 sheddase activity would require interaction with externalized PS, we used the soluble PS headgroup OPS that should act as competitive inhibitor in contrast to the PC headgroup OPC. In a first approach, sheddase function was determined in COS7 cells that were transfected with the AP-tagged ADAM10 substrate BTC. Induction of calcium influx e.g. by ionomycin (IO) is the classical pathway leading to PS externalization (Suzuki et al., 2010) and to ADAM10 activation (Maretzky et al., 2015). Accordingly, IO-treatment induced PS exposure (Supplementary Figure S1A) and BTC shedding in COS7 cells (Figure 1A). BTC shedding was significantly inhibited by broad-spectrum metalloprotease inhibitor TAPI-1, the preferential ADAM10 inhibitor GI254023X (GI) (Ludwig et al., 2005), and dose-dependently reduced by OPS (Figure 1A). A slight but not significant reduction was also observed for OPC. While OPS has two negative charges, OPC has one, due to the missing backbone probably providing the latter with some inhibitory potential.

Purinergic P2 receptor (P2R) activation is another important trigger of ADAM-mediated shedding (Pupovac and Sluyter, 2016). Stimulation of P2X receptors also activates Anoctamin-6 dependent PS scrambling (Ousingsawat et al., 2015). Cells were stimulated with melittin, a P2R activator and potent trigger of ADAM sheddase function (Sommer et al., 2012). Melittin stimulation led to a significant increase in ADAM10-mediated BTC release (Figure 1B) and PS externalization (Supplementary Figure S1B). Shedding was dose-dependently reduced by OPS, but not by OPC, and abrogated in the presence of the ADAM10 inhibitor GI.

We next analysed the release of AP-tagged VE-cadherin, an ADAM10 substrate (Schulz et al., 2008) that is structurally distinct from EGFR ligands. IO-induced VE-cadherin shedding was also significantly reduced in the presence of OPS, but not OPC (Figure 1C). Shedding was naturally inhibited by the broad-spectrum metalloprotease inhibitor TAPI-1 and the preferential ADAM10 inhibitor GI.

E-cadherin shedding (Maretzky et al., 2005) was analysed in HaCaT keratinocytes in order to extend the results to an endogenously expressed ADAM10 substrate. Ionomycin stimulation of HaCaTs in the presence of 10 mM OPS or OPC yielded results comparable to the previous data. OPS but not OPC suppressed shedding of the substrate as evidenced by immunoblot and densitometric analysis (Figure 1D and E).

### Rabbit erythrocytes as model for PS-dependent ADAM10 sheddase activation

**Figure 2:**
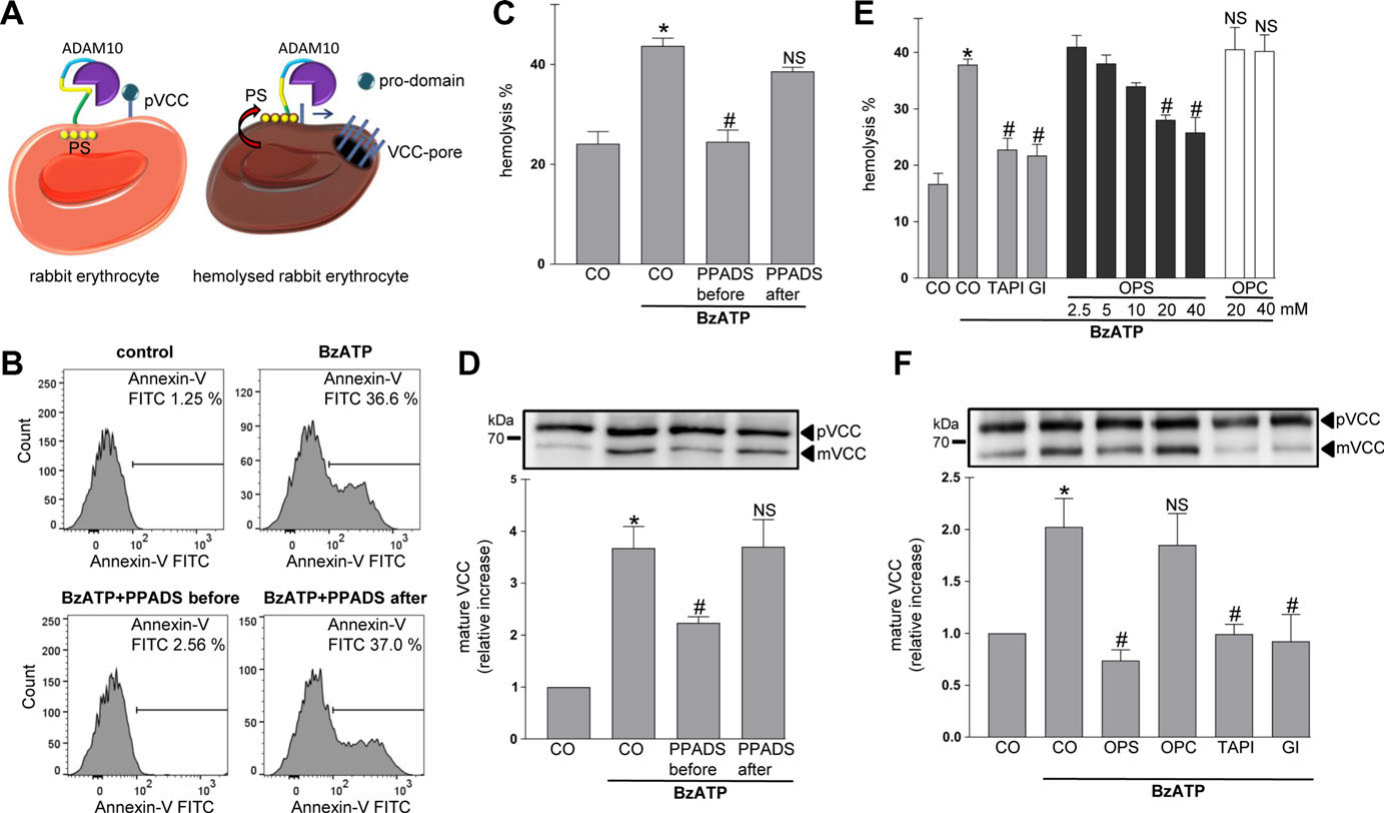
Analysis of ADAM10 PS-dependency using a rabbit erythrocyte model. (**A**) Schematic model. ADAM10 is expressed on rabbit erythrocytes. PS is externalized upon cell activation. *Vibrio cholerae* cytolysin precursor (pVCC) cleavage leads to pore formation and hemolysis. (**B**) Stimulation with BzATP (0.5 mM) leads to PS exposure. Cells were stimulated for 15 min, stained with Annexin V-FITC, and analysed by FACS analysis. Induction of PS exposure was inhibited by pre-incubation with P2R antagonist PPADS (100 μM), but not by post-incubation. (**C**–**F**) BzATP-induced hemolysis and pVCC cleavage depends on PS externalization. Cells were stimulated with BzATP as detailed in Materials and methods. BzATP treatment induced hemolysis (**C**) and pVCC cleavage (**D**). Pre-incubation with PPADS abrogated this effect. Application of PPADS after removel of BzATP could not prevent pVCC shedding and hemolysis. (**D**, upper panel) Representative immunoblot of pVCC cleavage. (**D**, lower panel) Densitometric quantification of three independent experiments. (**E** and **F**) BzATP-induced hemolysis (**E**) and pVCC cleavage (**F**) were inhibited by OPS (20 mM) but not by OPC (20 mM) and significantly abrogated in the presence of TAPI-1 (10 μM) and GI (3 μM). (**F**, upper panel) Representative immunoblot of pVCC cleavage. (**F**, lower panel) Densitometric quantification of three independent western blots. * indicates a significant increase compared to unstimulated cells; # indicates significant decrease compared to stimulated control cells (*P* < 0.05, *n* = 3; ±SEM). NS, no significant difference. Data were analysed by one-way analysis of variance and Bonferroni multiple comparison *post hoc* test. CO, control.

Rabbit erythrocytes were then employed to test the relevance of PS for ADAM10 function in a very simplified model system. ADAM10 is permanently exposed at the surface of these cells that are devoid of membrane traffic (Reiss et al., 2011). Sheddase activity can be assessed by planting an artificial substrate, pro-*Vibrio cholera* cytoloysin (pVCC) on the cell surface (Reiss et al., 2011). Cleavage of pVCC generates mature cytolysin (mVCC) (Figure 2A). The process is dually quantifiable by Western blot detection of cleaved toxin and by hemolysis measurements.

PS exposure in erythrocytes can be provoked by purinergic receptor activation (Sluyter et al., 2007). Cells were stimulated with BzATP and analysed for PS exposure by flow cytometry. As shown in Figure 2B, a subpopulation of cells exposed PS after 15 min. This effect could be inhibited by pre-incubation with the broad-spectrum P2R antagonist PPADS (Figure 2B). When PPADS was applied after stimulation, the antagonist could not prevent PS externalization. pVCC cleavage and hemolysis were markedly increased upon BzATP stimulation (Figure 2C and D). Pre-incubation with PPADS abrogated this effect. Cells were stimulated with BzATP in the presence of the broad-spectrum metalloproteinase inhibitor marimastat (MM) in order to provoke PS externalization while preventing substrate cleavage. Thereafter, inhibitor and stimulus were washed away and PS-positive cells were post-incubated in the presence of PPADS. Then, in the absence of any further stimulation, pVCC cleavage and hemolysis of the PS-positive cells took place and this could not be prevented by application of PPADS (Figure 2C and D). To verify that BzATP-induced pVCC cleavage and hemolysis were ADAM10 and PS-dependent, erythrocytes were stimulated in the presence of the respective inhibitors. As shown in Figure 2E and F, hemolysis and pVCC shedding were significantly reduced not only in the presence of the broad-spectrum metalloproteinase inhibitor TAPI-1 and the more specific ADAM10 inhibitor GI, but also by OPS (Figure 2E and F). The findings underlined the essential involvement of ADAM10 in the cleavage of pVCC and quite directly demonstrated the importance of PS exposure for triggering sheddase function.

### COS7 cells overexpressing ANO6 show increased PS exposure and increased ADAM10 sheddase activity upon calcium influx

**Figure 3:**
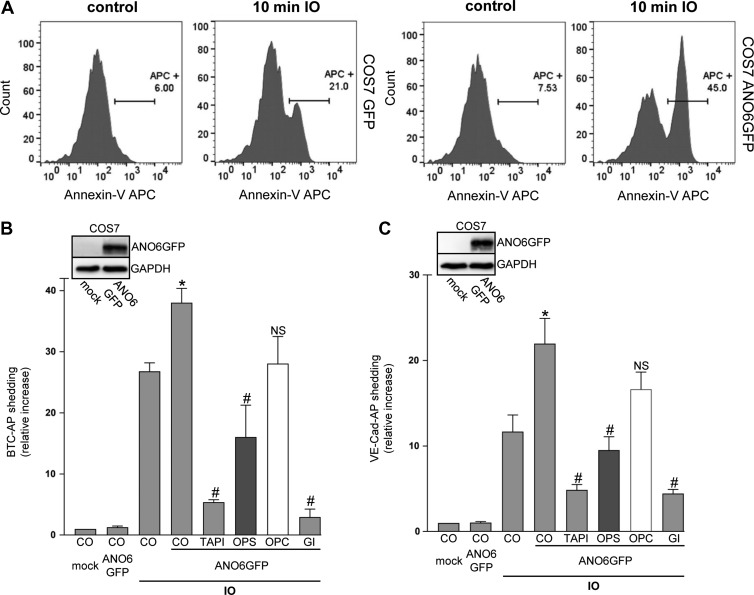
Overexpression of ANO6 enhances ADAM10 sheddase activity upon calcium influx. (**A**) Mock-transfected and Anoctamin-6 (ANO6)-GFP-transfected COS7 cells were stimulated with ionomycin (IO, 1 μM) for the indicated time. After stimulation, cells were stained with Annexin V-APC and analysed via FACS analysis. (**B** and **C**) COS7 cells were co-transfected with ANO6-GFP or mock vector and the AP-tagged ADAM10 substrates BTC or VE-cadherin, respectively. Cells were stimulated with IO (1 μM) for 30 min. IO-induced shedding was significantly increased upon overexpression of ANO6. TAPI (10 μM), ADAM10 inhibitor GI (3 μM), and OPS (10 mM), but not OPC (10 mM), significantly abrogated the induced shedding. * indicates a significant increase compared to mock-transfected stimulated cells; # indicates significant decrease compared to ANO6-transfected stimulated control cells (*P* < 0.05, *n* = 3; ±SEM). NS, no significant difference. Data were analysed by one-way analysis of variance and Bonferroni multiple comparison *post hoc* test. CO, control.

Anoctamin-6 (ANO6) has been identified as Ca^2+^-activated scramblase (Suzuki et al., 2010; Segawa and Nagata, 2015), and its overexpression reportedly led to increased PS exposure upon increase of cytosolic calcium levels (Yu et al., 2015). In accord with this, transfection of GFP-tagged ANO6 in COS7 cells led to an increase in the number of Annexin V-positive cells upon ionophore stimulation (Figure 3A and Supplementary Figure S2A).

When COS7 cells were co-transfected with GFP-tagged ANO6 and BTC-AP, ANO6 overexpression significantly increased ionomycin-induced BTC shedding compared to mock-transfected cells (Figure 3B). This increase was abrogated in the presence of TAPI, the ADAM10 inhibitor GI, OPS, but not OPC. Comparable data were obtained for VE-cadherin shedding (Figure 3C). In addition to the optical control, anti-GFP western blots confirmed ANO6 transfection in each experiment (inserts Figure 3B and C).

### A constitutively active form of ANO6 increases BTC and VE-cadherin shedding in the absence of any stimulus

**Figure 4:**
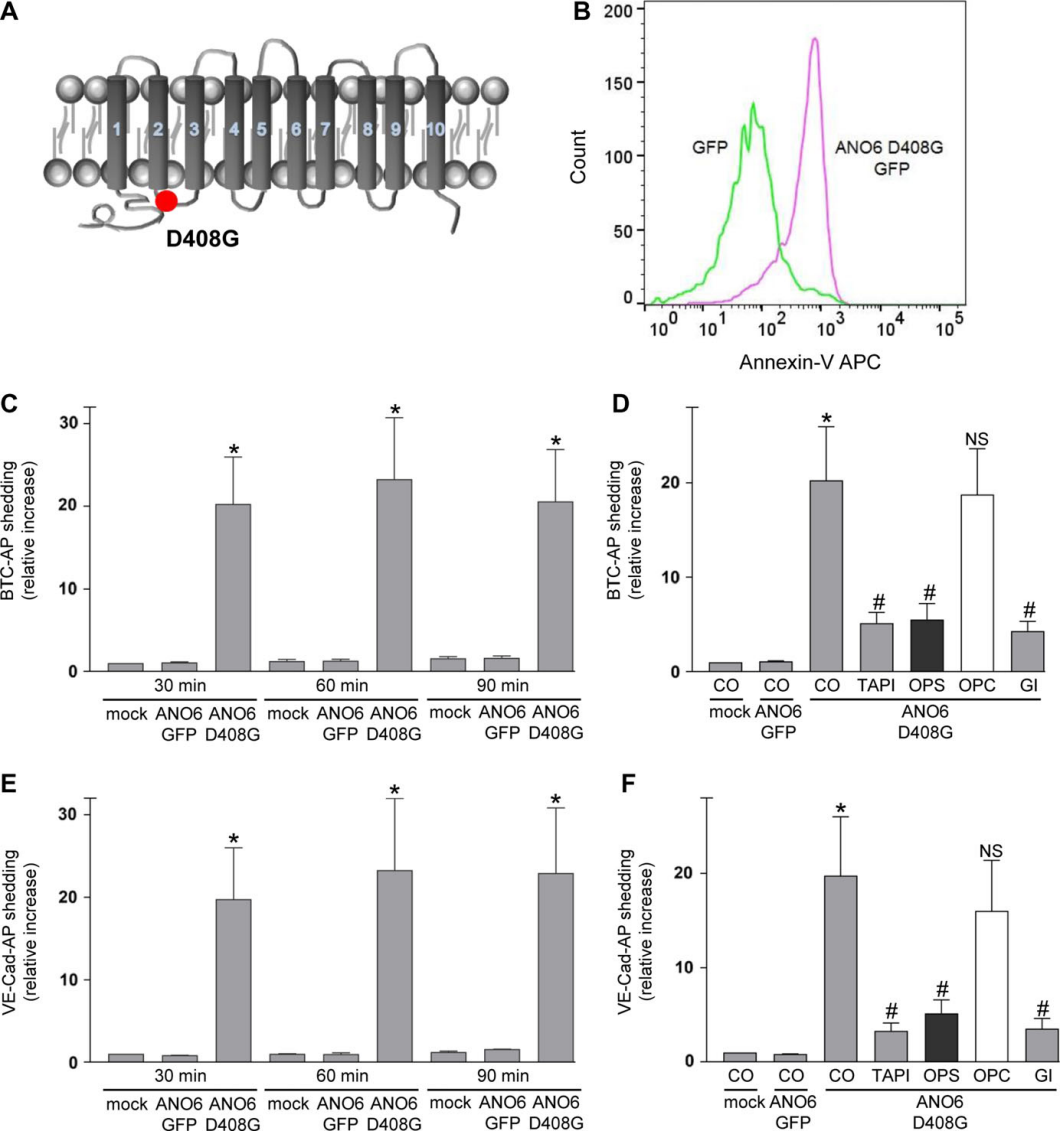
Transfection of hyperactive ANO6 leads to constitutive PS exposure and ADAM10 sheddase activation. (**A**) Schematic representation of human ANO6-D408G mutant. (**B**) Mock-transfected or ANO6-D408G-GFP-transfected COS7 cells were analysed for PS exposure using Annexin V-APC staining. (**C**–**F**) COS7 cells were co-transfected with mock plasmid or ANO6-D408G-GFP and the AP-tagged ADAM10 substrate BTC (**C**, **D**) or VE-cadherin (**E**, **F**). (**C** and **E**) After washing, cells were analysed for substrate release for 30, 60, and 90 min. (**D** and **F**) Cells were analysed for BTC-AP or VE-cadherin release after 30 min in the presence of TAPI-1 (10 μM), OPS (10 mM), OPC (10 mM), or GI (3 μM). ADAM10 inhibitor GI, TAPI, and OPS significantly abrogated the shedding. * indicates a significant increase compared to mock-transfected cells; # indicates significant decrease compared to ANO6-D408G-GFP transfected cells in the absence of inhibitor (*P* < 0.05, *n* = 3; ±SEM). NS, no significant difference. Data were analysed by one-way analysis of variance and Bonferroni multiple comparison *post hoc* test. CO, control.

The key observation leading to the identification of ANO6 as Ca^2+^-activated scramblase was the constitutive exposure of PS at the surface of a mouse B-cell line. The cDNA was found to encode ANO6 harbouring a single point mutation (D409G, corresponding to human D408G; Figure 4A). This rendered the scramblase hypersensitive to intracellular Ca^2+^ levels (Suzuki et al., 2010; Kunzelmann et al., 2012). In a next experiment, we analysed the impact of this ANO6 mutant on PS externalization and on its influence on ADAM10-mediated shedding in COS7 cells. Transfection of GFP-tagged ANO6-D408G led to increased Annexin V staining in the absence of stimulation (Figure 4B and Supplementary Figure S2B). Upon co-transfection of this mutant with BTC-AP or VE-cadherin-AP, maximal constitutive substrate release was observed already within 30 min (Figure 4C and E). Longer incubation periods led to no further increase in the amount of soluble substrate. Inhibitor experiments confirmed that ADAM10 was responsible for the PS-dependent shedding of VE-cadherin and BTC (Figure 4D and F). These data provided a further line of evidence that ADAM10 sheddase function is triggered by PS in the outer membrane leaflet.

### PS externalization defects result in reduced ADAM10-mediated release of CD23

**Figure 5:**
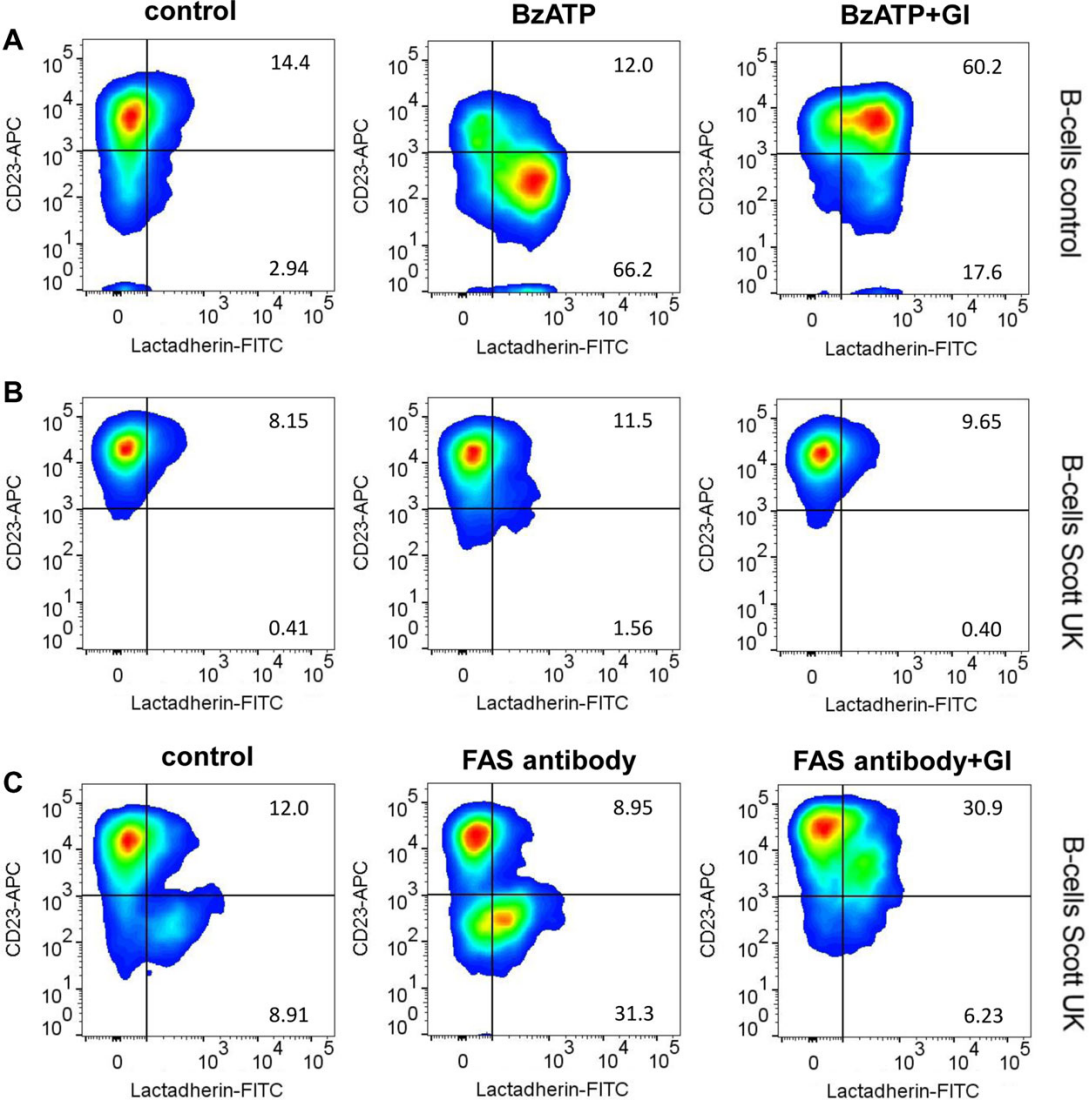
ANO6 loss-of-function abolishes ADAM10-mediated shedding in Scott patient lymphocytes. Control B-cells and B-cells from Scott syndrome patients were monitored by flow cytometry for induction of PS exposure (lactadherin-FITC) and CD23 shedding. (**A** and **B**) BzATP stimulation (0.5 mM, 30 min) induced PS externalization and loss of CD23 in control cells (**A**, middle panel), but not in Scott patient cells (**B**, middle panel). (**C**) Fas-antibody (500 ng/ml, 6 h)-induced apoptosis led to pronounced PS exposure and concomitant CD23 loss in Scott cells. BzATP and Fas-antibody-induced CD23 shedding was abrogated in the presence of the ADAM10 inhibitor GI (3 μM). Representative pseudocolor plots of three independent experiments are shown.

Loss of function mutations in ANO6 of hematopoietic cells lead to Scott syndrome, a rare bleeding disorder (Suzuki et al., 2010; Castoldi et al., 2011). ADAM10 has been described to act as the major sheddase of the low affinity IgE receptor CD23 on B-cells following purinergic receptor stimulation (Gu et al., 1998; Weskamp et al., 2006; Lemieux et al., 2007). Immortalized B-cells from a Scott syndrome patient were found to show the same surface expression of ADAM10 as control cells (Supplementary Figure S3) and a comparable expression of CD23 (Figure 5). Normal B-cells and Scott cells were then monitored by flow cytometry for induction of CD23 shedding. PS exposure was visualized using FITC-labelled lactadherin. When BzATP was applied to control B-cells, flow cytometric analyses revealed rapid PS exposure that was paralleled by loss of CD23 from the cell surface (Figure 5A, centre panel and Supplementary Figure S4). CD23 shedding was abrogated in the presence of the ADAM10 inhibitor GI (Figure 5A, right panel). Scott lymphocytes responded to BzATP neither with PS exposure nor with shedding of the ADAM10 substrate (Figure 5B and Supplementary Figure S4). However, apoptosis induction with Fas-antibody revealed that these cells were able to translocate PS most likely via activation of apoptosis-sensitive scramblases, and CD23 was then shed in an ADAM10-dependent manner (Figure 5C).

### The ADAM10 stalk region interacts with PS- but not with PC liposomes

**Figure 6:**
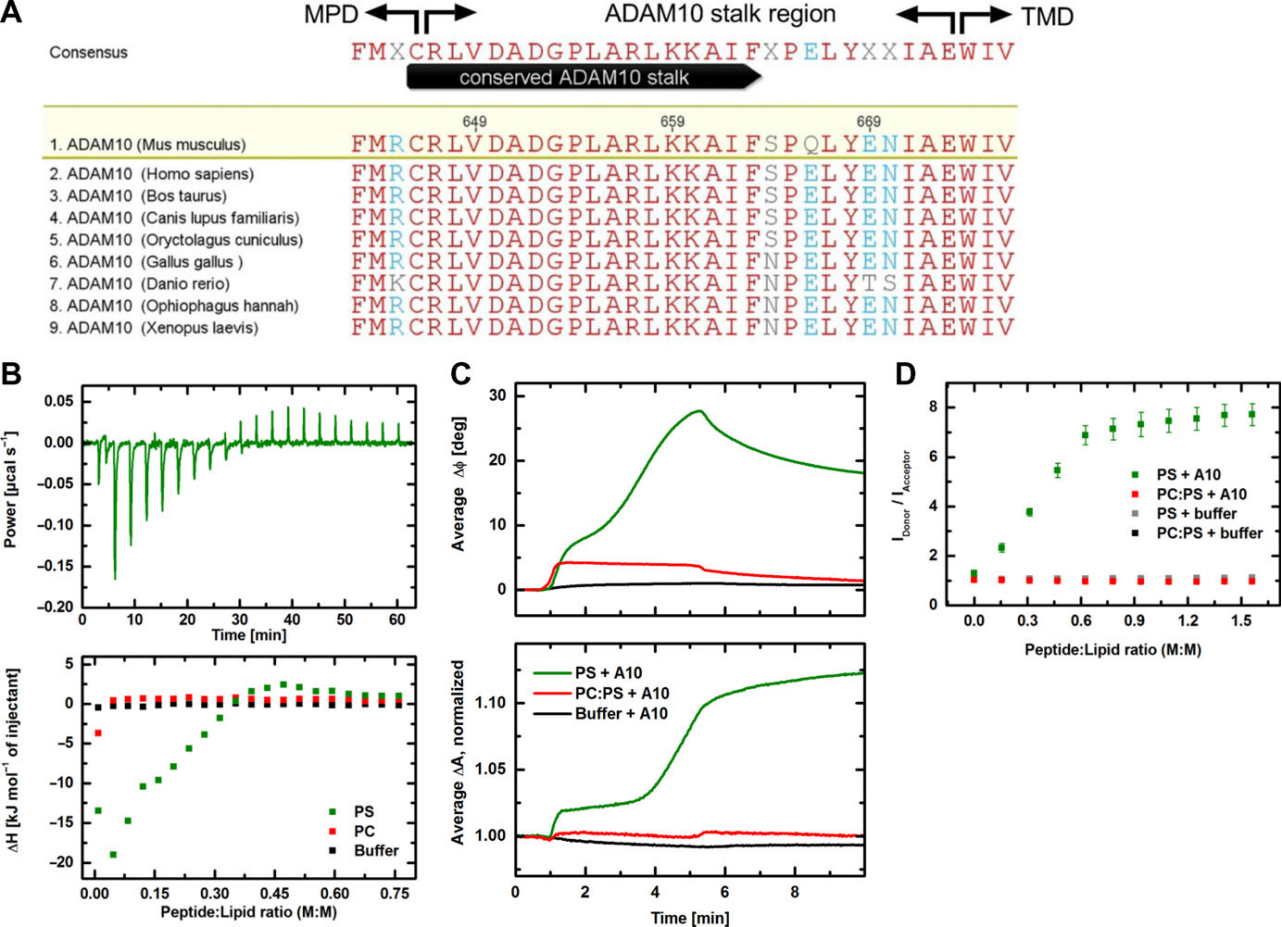
Biophysical properties of the ADAM10 stalk region. (**A**) Alignment of the ADAM10 stalk region sequences downstream of the membrane proximal domain (MPD) and upstream of the transmembrane domain (TMD) from different species identifies a highly conserved region. Red letters indicate identical residues. ClustalW multiple sequence alignment. (**B**) ITC measurements to study the interaction of the ADAM10 stalk region with either PS (green) or PC (red) liposomes. Shown is the heating power corresponding to interaction of the ADAM10 peptide with PS liposomes in 20 titration steps as a function of time (upper panel) and the interaction enthalpy for both lipid systems and the buffer control (black) plotted over the peptide:lipid ratio (lower panel). While the titration of ADAM10 peptide into buffer and to PC liposomes shows no interaction, it comes first to exothermic reactions and then to endothermic reactions with PS liposomes. (**C**) SAW measurements showing the interaction of ADAM10 peptide with PS membranes (green) or PC:PS (9:1) membranes (red) immobilized on the CM-dextran/poly-L-lysine (PLL) functionalization of the sensor chip surface. Binding of the peptide to the functionalization (black) served as control. The increase of the phase signal ΔΦ indicates an additional mass loading on the chip surface being more pronounced for PS than for PC:PS (upper panel); the increase of the amplitude signal ΔA corresponds to an increased viscosity on the surface (lower panel). Shown are average curves of five individual sensor channels. (**D**) Fluorescence resonance energy transfer (FRET) spectroscopy. Intercalation of ADAM10 peptide into PS liposomes (green) and PC:PS (9:1) liposomes (red). The liposomes are double labelled with NBD-PE (donor) and Rhodamine-DHPE (acceptor). An increased ratio between donor and acceptor fluorescence intensities indicates insertion of peptides between the lipid molecules. This effect is only visible for PS liposomes but not for PC:PS liposomes. Controls (grey and black) show no effect. Error bars indicate the standard deviations of three independent measurements.

Thus far, the data tied in with the notion that, similar to ADAM17, ADAM10 sheddase activation was PS-dependent. The question remained whether any mechanistic similarities with ADAM17–PS interaction would exist. ADAM17 has two putative membrane interaction sites: the cationic binding motif in the MPD and the amphipathic CANDIS region. Examination of the amino acid sequence disclosed no cationic motif in the ADAM10 MPD. However, a candidate motif similar to that identified in the ADAM17 MPD was present in the evolutionarily conserved stalk region (Figure 6A) of ADAM10. Therefore, experiments were undertaken with the isolated stalk region to investigate whether interaction with membrane PS might be detectable.

In isothermal titration calorimetry (ITC) experiments, an exothermic reaction (negative enthalpy value ΔH) was observed upon the titration of ADAM10 peptide into PS liposome solution. This reaction went into saturation during the titration and was superimposed by a delayed endothermic reaction (positive enthalpy values). On the other hand, no reaction could be detected using PC liposomes (Figure 6B). Titration of a control peptide (KT peptide) with scrambled sequence instead of the ADAM10 peptide to PS liposomes resulted in an exothermic reaction with saturation at lower peptide-lipid ratio with no additional endothermic reaction (Supplementary Figure S5). This qualitatively different effect shows that the interaction between ADAM10 peptide and PS is not only mediated by the positive charge, but also by the unique primary structure of the peptide.

Surface acoustic wave (SAW) experiments employing biosensor chips coated with either PC or PC:PS (9:1, M:M) membranes revealed that the ADAM10 peptide bound to PS membranes in a two-step process (Figure 6C, upper panel) and thereby also led to a viscosity increase of the membrane which could be indicative of peptide intercalation (Figure 6C, lower panel). Compared to PS membranes, the binding to PC:PS (9:1) membranes was only moderate and no change of the viscoelastic properties was detectable. Also in this case, a clear influence of the primary structure on the qualitative course of the interaction could be shown by experiments with the control peptide (Supplementary Figure S5). In contrast to the ADAM10 peptide, a distinct binding of the KT peptide was accompanied by a significantly reduced viscosity of the membrane.

The findings were corroborated by FRET spectroscopy which confirmed that the peptide became intercalated into PS liposomes, but not in PC:PS (9:1) liposomes (Figure 6D). In this measuring system, the intercalation of the control peptide changed into a precipitation of the reactants (Supplementary Figure S5).

**Figure 7:**
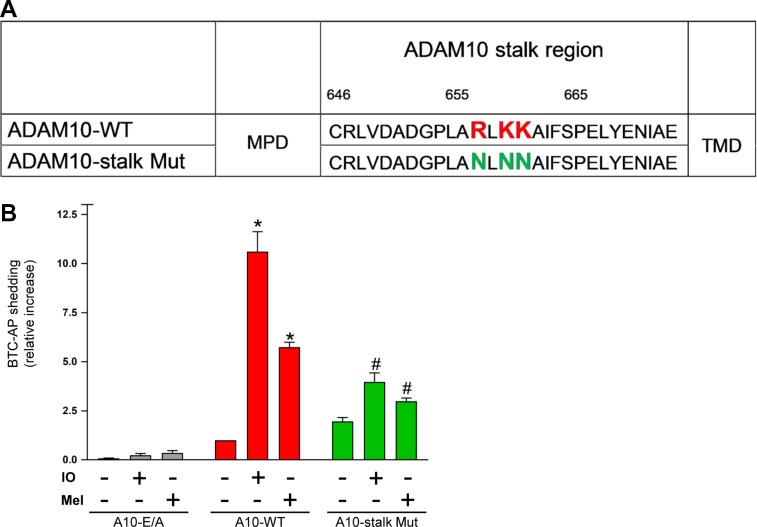
Deletion of cationic amino acids in the ADAM10 stalk region impairs sheddase function. (**A**) A potential PS-binding motif (R657/K659/K660) was exchanged creating an ADAM10 stalk mutant (ADAM10-stalk Mut). (**B**) ADAM17/ADAM10 double-deficient HEK cells were co-transfected with BTC-AP and WT-ADAM10 (A10-WT), inactive ADAM10 (A10 E/A), or ADAM10 mutant and stimulated with ionomycin (IO, 1 μM) or melittin (Mel, 1 μM) for 30 min. AP-activity in the supernatant was calculated in relation to total (supernatant and cell pellet) AP activity and is shown in comparison to A10-WT as ‘control’. * indicates a significant increase compared to unstimulated cells; # indicates significant decrease compared to A10-WT transfected stimulated cells (*P* < 0.05, *n* = 3; ±SEM). Data were analysed by one-way analysis of variance and Bonferroni multiple comparison *post hoc* test.

To assess the relevance of the triplet cationic amino acid cluster in the stalk region, an ADAM10 mutant was constructed in which asparagines replaced R657/K659/K660 (A10-stalk Mut, Figure 7A). ADAM10 and ADAM17 double-deficient HEK cells (Riethmueller et al., 2016) were used for transfection experiments in order to completely avoid any background. The cells were co-transfected with AP-tagged BTC and wild-type (WT)-ADAM10 or the enzymatically inactive ADAM10-E/A, or the ADAM10 stalk mutant. As shown in Figure 7B, shedding of BTC was induced by IO and melittin in cells transfected with WT-ADAM10 but not with the enzymatically inactive ADAM10-E/A. Stimulated shedding was significantly reduced in cells transfected with the ADAM10 stalk mutant. Western blot and flow cytometric analysis disclosed that lack of sheddase activity was not due to reduced protein expression, maturation, or cell surface localization of the mutant (Supplementary Figure S6).

## Discussion

The objective of this investigation was to examine whether PS exposure might play a relevant role in regulating sheddase activity of ADAM10, as had previously been discerned for ADAM17 (Sommer et al., 2016b). In the latter investigation, stimulated sheddase activity was found to correlate with and depend on surface-exposure of PS. This was demonstrated in different cell models using a spectrum of sheddase triggers.

In the case of ADAM10, the universally reliable means of enhancing shedding function is through induction of Ca^2+^ influx, as conventionally provoked with the use of Ca^2+^-ionophores. In the first set of experiments, cells were exposed to ionomycin and ADAM10 sheddase function was quantified in the presence or absence of phosphorylserine or phosphorylcholine, the soluble head groups of PS and PC, respectively. COS7 cells transfected with two different ADAM10 substrates served as one model, and cleavage of the endogenous substrate E-cadherin in HaCaT was assessed in a second system. In all cases, it was found that ionomycin-mediated enhancement of substrate cleavage was dose-dependently reduced in the presence of OPS but not of OPC. This was interpreted to indicate that OPS specifically interfered with the shedding process, presumably by competing in the event with surface-exposed PS.

Cytosolic Ca^2+^ elevation has long been known to provoke PS exposure. However, a candidate molecule that would affect PS-translocation has only quite recently been discovered. Anoctamin-6 is a Ca^2+^-activated ion channel that simultaneously functions as a scramblase, rapidly shuttling PS from the internal to the outer membrane leaflet. Two major findings followed in the wake of this discovery that proved invaluable in our present work. First, a single amino acid substitution leads to gain of scramblase function in the absence of Ca^2+^ influx (Suzuki et al., 2010). Second, another mutation leads to loss of scramblase function. The latter is the cause of a rare bleeding disorder, Scott syndrome, because of the inability of activated platelets to externalize PS (Castoldi et al., 2011). Through employment of these mutants, it became possible to very specifically address the significance of PS exposure for ADAM10 function.

An exploratory investigation into the effects of ANO6-overexpression was first undertaken. COS7 cells were co-transfected with a combination of ANO6-GFP and the sheddase substrates BTC-AP or VE-cadherin-AP. ANO6 overexpression resulted in enhanced PS exposure upon cell stimulation with ionomycin. This was associated with augmented ADAM10 sheddase activity that was again specifically inhibited by OPS.

The human D408G gain-of-function mutant was then employed. COS7 cells transfected with this mutant scramblase spontaneously hyperexposed PS without any requirement for stimulation. This finding accorded perfectly with the original observation that had led to discovery of the murine D409G mutant (Suzuki et al., 2010). The B-cell line harbouring the mutation was detected because of the conspicuous, constitutive exposure of PS on the surface of the cells (Suzuki et al., 2010).

Co-transfection with AP-tagged ADAM10 substrates then generated intriguing findings. Constitutive cleavage of both BTC and VE-cadherin by ADAM10 rose to remarkable levels, comparable to those normally observed upon ionophore stimulation. Again, sheddase activity was efficiently suppressed in the presence of extracellular OPS. These findings were particularly informative because no measure further to the introduction of the hyperactive scramblase with its PS-translocating property was required to spontaneously increase sheddase activity. And because this in turn could be suppressed by OPS, the conclusion appeared inescapable that PS exposure was indeed the key factor responsible for the effect.

Experiments with B-cells harbouring a loss-of-function ANO6 mutant complemented the above findings. ADAM10 function was assessed by flow cytometric analysis of CD23 shedding from the cells. In normal B-cells, application of BzATP led to rapid PS externalization and shedding of CD23. Both reactions were absent in Scott lymphocytes harbouring the loss-of-function ANO6 mutant. The findings accorded with the notion that purinergic receptor agonists provoke downstream triggering of Ca^2+^-activated channels, that ANO6 is the central scramblase mediating P2R-induced PS externalization (Taylor et al., 2008; Ousingsawat et al., 2015), and that the latter event is instrumental in upregulating ADAM10 sheddase function.

The pivotal role played by PS was borne out in the next set of experiments utilizing an entirely different model. Rabbit erythrocytes express ADAM10 on their surface (Reiss et al., 2011). Sheddase activity can easily be assessed by planting an artificial substrate pro-VCC on the cell surface. Cleavage of the pro-toxin generates the mature pore-forming cytolysin that is detectable both by western blotting and by virtue of its haemolytic activity. Erythrocytes harbour purinergic receptors and ANO6 (Sluyter, 2015; Bevers and Williamson, 2016), so the following experiments with an important built-in feature became possible in which PS-translocation was temporally dissociated from the proteolytic event. BzATP was first applied in the presence of the reversible ADAM inhibitor TAPI-1. This led to PS exposure that was abrogated if cells were pre-treated with the purinergic receptor antagonist. As to be expected, post-treatment with the antagonist did not reverse PS exposure once the event had taken place. In the second step, the protease inhibitor was removed and the ensuing substrate cleavage monitored. The results clearly showed that cleavage was enhanced in cells exposing PS. This was not dependent on continued purinergic receptor signalling and persisted even if the receptor antagonist was applied thereafter. The central role of surface-exposed PS in upregulating sheddase function in this simple model was again underlined by the finding that cleavage of the pro-toxin could be suppressed by soluble OPS.

In the previous investigation, a triplet cluster of cationic amino acids RK_K was identified as the functionally important PS-binding motif positioned within the MPD and immediately adjacent to the stalk region (CANDIS) of ADAM17 (Sommer et al., 2016b). A similar motif R657/K659/K660 was observed to be present in the stalk region of ADAM10, which was recently observed to bind to PS liposomes (Dusterhoft et al., 2015). In extension of that investigation, ITC revealed an interaction of the peptide with PS but not with PC liposomes. An initial exothermal reaction indicative of association of the peptide with the membrane was followed by an endothermal reaction that could reflect a conformational change undergone by the lipid-bound peptide. This contention received support from data obtained with SAW biochip analyses in which sensor chips were coated with either PS or PC:PS membranes. A two-step binding process was observed only with PS membranes. The second binding step may have corresponded to the endothermic reaction observed in the ITC experiments. The viscosity increases occurring during peptide interaction with PS membranes might be indicative of an intercalation of the peptide into the lipid bilayer. Spectroscopic FRET measurements were also indicative of an insertion of the peptide between the lipid molecules of pure PS liposomes. Collectively, the biophysical analyses showed that PS exposure plays a relevant role for the stalk peptide-membrane interaction. The data indicate that the conserved ADAM10 stalk region might unite the biophysical properties that in ADAM17 are separately located in the MPD and CANDIS region.

Upon removal of the cationic motif, the mutated protease continued to be expressed in its mature form and exposure at the cell surface was ascertained by immunoblot and flow cytometry. However, the mutant ADAM10 showed significantly reduced shedding capacity upon ionomycin or melittin stimulation.

All our data would be in line with a concept that PS translocated to the outer membrane leaflet is pivotal for ADAM10 to exert its sheddase function. Interestingly, the striking effects of PS externalization are not limited to ADAM10. As we have shown previously, ADAM17 sheddase function also relies on transient breakdown of phospholipid asymmetry. It will be of high interest to delineate the differences and the similarities that might depend not only on the nanodomain localization, the substrate availably, but also on the type of stimulus and the respective scrambling machinery that is activated. Thus, the classical ADAM17 activator PMA also induces PS exposure without activating ADAM10, while ionomycin can principally activate both proteases.

**Figure 8:**
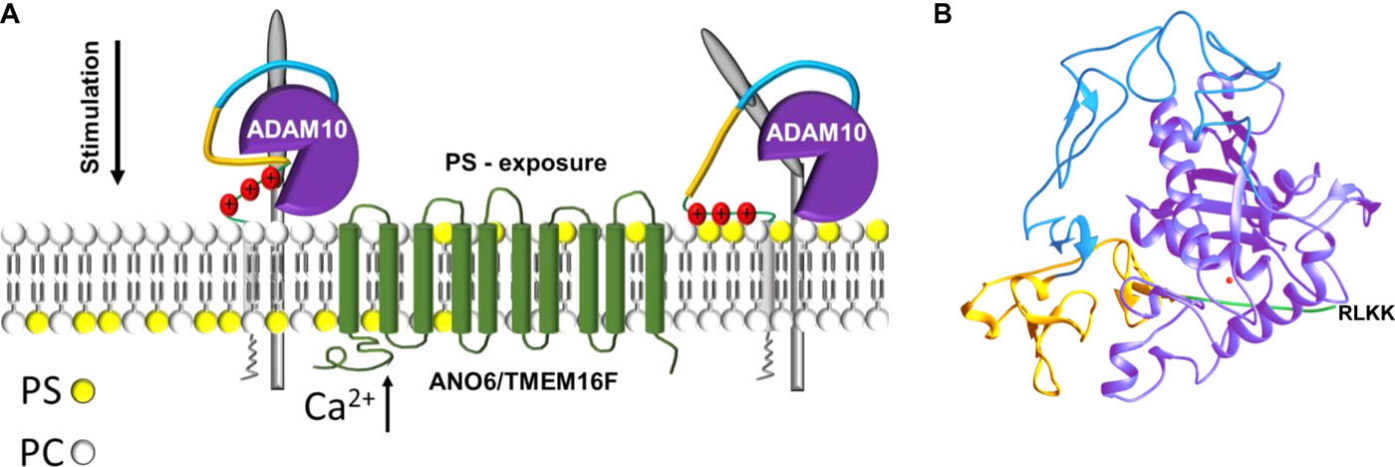
Model of ADAM10 sheddase activation. (**A**) Stimuli leading to elevation of intracellular calcium induce ANO6 scramblase activation and PS externalization from the inner to the outer cell membrane leaflet. Hyperactive ANO6 induces ADAM10-mediated shedding even in the absence of any stimulus. Cationic amino acid residues in the stalk region of ADAM10 might interact with the negatively charged PS-head group. ADAM10–PS interaction seems to be a prerequisite for induced shedding. (**B**) Ribbon representation of the X-ray structure of the extracellular region of ADAM10 (pdb accession code: 6BE6) (Seegar et al., 2017). Colour coding of the different domains is according to **A** and the Zn-ion in the catalytic centre is depicted in red. Cationic amino-acid residues in the stalk region responsible for the PS interaction are missing in the X-ray structure and are depicted in one-letter code.

Most interestingly, the 3D structure of the ADAM10 extracellular domain has recently been reported (Seegar et al., 2017). It was found that the enzyme active site is occluded by a short peptide loop located at the commencement of the stalk region (residues 647–655). The putative PS-binding motif follows immediately after this inhibitory loop (residues 657/659/660). Attraction of the cationic motif to surface exposed PS may thus serve to draw this loop out of the catalytic site enabling substrate access (Figure 8A and B). Future work will show whether this straightforward model for the regulation of ADAM10 by the biological membrane is correct.

## Materials and methods

### Reagents and antibodies

Pyridoxalphosphate-6-azophenyl-2′,4′-disulfonic acid (PPADS), BzATP triethylammonium salt, TAPI-1, ADAM10 inhibitor GI254023X (Ludwig et al., 2005), marimastat, and ionomycin were obtained from Tocris. Annexin V-APC and Annexin V-Alexa 568 were from Thermo Fisher Scientific. Lactadherin-FITC was purchased from Haematologic Technologies. *Vibrio cholerae* pro-cytolysin and polyclonal rabbit antisera against VCC were produced as described (Valeva et al., 2004). Monoclonal antibodies against the cytoplasmic domain of E-cadherin (C36) were purchased from BD Bioscience. N-terminal and C-terminal antibodies against ADAM10 were from Abcam. Anti-GAPDH (1:500) was from Santa Cruz Biotechnology. Secondary POD antibodies were purchased from Dianova. Phosphorylserine (O-phospho-L-serine, OPS), 4-(2-Aminoethyl)benzenesulfonyl fluoride hydrochloride (AEBSF), complete, and PhosStop were purchased from Sigma-Aldrich. Phosphocholine (OPC) was obtained from Tokyo Chemical Industry. PS and PC were from Avanti Polar Lipids. Melittin was synthesized as described (Andra et al., 2004). ADAM10 stalk region (CRLVDADGPLARLKKAIFSGSGSGK) and a control peptide with scrambled sequence (DRGALLICSFDAVLKRPAKGSGSGK) were produced by Biosyntan. Antibodies for flow cytometric analysis of human ADAM10 were purchased from R&D and for murine ADAM10 from Abcam. Secondary antibody anti-rabbit Alexa Fluor 488 was from Thermo Fisher Scientific. Additional antibodies used for flow cytometry were as follows: CD23 (BD Bioscience, 1:100), IgG1-APC (BD Bioscience, 1:50), anti-Fas Antibody CH11 (Merck Millipore). NBD-phosphatidylethanolamine (NBD-PE; REF: N360) and Lissamine rhodamine B (Rhodamine-DHPE; REF: L1392) were purchased from Molecular Probes (Thermo Fisher Scientific).

### Cell culture

HaCaT cells were provided by Dr N.E. Fusenig (DKFZ, Heidelberg, Germany) (Boukamp et al., 1988). ADAM10/ADAM17 double-deficient HEK-293T cells have been described before (Riethmueller et al., 2016). HaCaT, HEK, and COS7 cells (ATCC) were grown in high glucose DMEM (Thermo Fisher Scientific) supplemented with 10% foetal calf serum (FCS) and 1% penicillin/streptomycin (Pen/Strep). EBV-transformed B lymphoblast cell lines from control subjects and a Scott UK patient have been described before (Williamson et al., 2001; Munnix et al., 2003). Lymphocytes were grown in RPMI-1640 medium (GIBCO) supplemented with 10% FCS (GIBCO) and 1% Pen/Strep.

### Expression vectors

The expression vector for human ANO6-WT-GFP was kindly provided by Karl Kunzelmann. All mutations were inserted using the Quikchange II site-directed mutagenesis kit (Agilent Technologies). The kit was used according to the manufacturer’s instructions. The primer sequences for ANO6 D408G point mutation were 5′-GAACTTGAGTATGAATGGGGCACTGTTGAGTTACAGCAGG-3′ (fwd) and 5′-CCTGCTGTAACTCAACAGTGCCCCATTCATACTCAAGTTC-3′ (rev). The primer sequences for the ADAM10 stalk mutant (R657N, K659N, and K660N) were 5′-GATGGCCCTCTAGCTAATCTGAAAAAAGCC-3′ (fwd), 5′-GGCTTTTTTCAGATTAGCTAGAGGGCCATC-3′ (rev), 5′-CCTCTAGCTAATCTGAACAACGCCATTTTTAG-3′ (fwd), 5′-CTAAAAATGGCGTTGTTCAGATTAGCTAGAGG-3′ (rev).

Successful mutation was verified by sequencing. The plasmids for alkaline phosphatase (AP)-tagged BTC und VE-cadherin expression were from Dr Carl P. Blobel (Hospital for Special Surgery, New York, USA).

### Transfection and AP-substrate shedding assay

COS7 cells or ADAM10/ADAM17 double-deficient HEK-293T cells were transfected in 12-well plates using Turbofect Transfection Reagent (Thermo Fisher Scientific) according to the manufacturer’s instructions. A GFP expression plasmid (pcDNA3.1-GFP) was used as mock control for transfection experiments with ANO6-GFP or ANO6-D408G-GFP and pcDNA vector was used as a control for WT-ADAM10 or ADAM10 stalk mutant BTC-AP-shedding experiments. Twenty-four hours after transfection, cells were washed with DMEM which was replaced by fresh serum-free DMEM and pre-incubated with the indicated concentrations of TAPI-1, OPS, OPC, or GI for 15 min. Thereafter, cells were treated or not treated with ionomycin (1 μM, 30 min) or melittin (0.5 μM, 30 min for COS7 or 1 μM, 30 min for HEK-293T). Supernatants and cell lysates were collected and measured for AP activity at A405 nm with sunrise photometer (TECAN) employing the AP substrate 4-nitrophenyl phosphate (Sigma-Aldrich). Shown is the relative AP activity in the supernatant compared to the total AP activity of supernatant plus cell lysates normalized to the unstimulated cells.

### Detection of surface exposed PS

PS externalized to the outer cell membrane surface was detected by staining with fluorescent-labelled Annexin V or lactadherin. The latter proved to have superior sensitivity (Otzen et al., 2012) and was employed to stain lymphocytes in the flow cytometric experiments (see below). COS7 cells were seeded on glass coverslips. After 24 h, cells were incubated for the indicated time period with or without ionomycin (1 μM) or melittin (0.5 μM). After indicated stimulation periods coverslips were immediately incubated with a 1:20 solution of Annexin V-Alexa 568 (Thermo Fisher Scientific) in Annexin V-binding buffer (ABB:10 mM HEPES, 140 mM NaCl, and 2.5 mM CaCl_2_, pH 7.4) for 10 min in the dark, washed twice with ABB and fixed for 30 min with 3% paraformaldehyde (PFA). After fixation, coverslips were washed twice with PBS, once with distilled water and mounted in embedding medium. Image acquisition was performed with an inverted confocal microscope (Fluoview FV1000, Olympus) using a UPLSAPO 60× oil immersion objective (NA:1.35). Images were acquired with the same laser and detection settings for each experimental setup. For the FACS analysis of PS externalization, COS7 cells were transfected with ANO6GFP or GFP control vector. Twenty-four hours after transfection cells were detached from culture dish with Accutase (GE Healthcare). The cells were transferred into serum free DMEM and incubated for 10 min with or without ionomycin (1 μM) and subsequently stained with Annexin V-APC (Thermo Fisher Scientific). ANO6 D408G-GFP or GFP control vector transfected COS7 cells were detached with Accutase 24 h post-transfection and stained in serum free DMEM with Annexin V-APC for flow cytometry analysis.

### Western blot analysis

Cells were grown to ~80% confluency. Cells were pre-incubated with indicated ADAM10-modulating agents for 15 min and afterwards stimulated with 1 μM ionomycin for 20 min at 37°C. After stimulation, cells were washed once with PBS and lysed with lysis buffer (5 mM Tris-HCl, pH 7.5, 1 mM EGTA, 250 mM saccharose, and 1 % Triton X-100) supplemented with cOmplete inhibitor cocktail, PhosStop, and 10 mM 1,10-phenantroline monohydrate. Equal amounts of protein were loaded on 10% SDS–PAGE gels. Samples were electrotransferred onto polyvinylidene difluoride membranes (Hybond-P; Amersham) over night. Blots were blocked for 1 h with 5% skim milk in Tris-buffered saline at room temperature (RT). After incubation with the respective antibody in blocking buffer, the membranes were washed three times in TBST (Tris-buffered saline containing 0.1% Tween-20). Primary antibodies were detected using peroxidase (POD)-conjugated secondary antibodies (1:10000) for 45 min at RT. Detection was carried out using the ECL detection system (Amersham). Equal loading was controlled using GAPDH antibody (1:500). Signals were recorded by a luminescent image analyzer (Fusion FX7 imaging system; PEQLAB Biotechnologie). E-cadherin signals were analysed with Bio1D software (Vilber).

### Flow cytometric analysis

For analysing ADAM10 cell surface expression, Scott patient B-lymphocytes and control B-lymphocytes were washed with NaCl-medium (145 mM NaCl, 5 mM KCl, 5 mM Glucose, 10 mM Hepes) and stained with anti-ADAM10 (1:100) or rabbit IgG-control antibodies (1:100) in 500 μl NaCl medium with 3% BSA for 30 min on ice. After washing, cells were incubated with anti-rabbit Alexa Fluor 488 (1:250) for 30 min on ice. Cells were washed and re-suspended in NaCl medium.

ADAM10/ADAM17 double-deficient HEK-293T were transfected with either WT-ADAM10 or ADAM10 stalk mutant and surface levels were analysed with ADAM10 N-terminal antibody (Abcam) or isotype control IgG antibody (1:100). Cells were fixed with 1% PFA for 10 min and washed before staining was performed. Cells were stained with the primary antibody for 1 h in PBS with 3% BSA and human FC block (BD Bioscience). After the washing, the cells were stained with secondary antibody anti-rat Alexa488 (1:500) for 30 min. Thereafter, cells were washed and resuspended in PBS. All incubation steps were carried out on ice.

For analyzing CD23 shedding, cells were stimulated with BzATP in the presence of lactadherin-FITC (8.53 nM) for 30 min at 37°C. Involvement of ADAM10 activity was analysed by pre-incubation (15 min) with the inhibitor GI (3 μM). Lymphocytes were washed once with NaCl medium and fixed with 1% PFA solution for 10 min. Fixed lymphocytes were stained with CD23-APC or control IgG for 30 min on ice and afterwards washed twice with NaCl medium. Staining after Fas-Ab treatment (6 h for Scott cells) was performed as follows: cells were stained with lactadherin-FITC in RPMI medium for 10 min in the dark at 37°C, washed with NaCl medium, and fixed for 10 min with 1% PFA at RT. Cells were subsequently washed in NaCl medium and stained for 30 min with the CD23-APC antibody at 4°C in NaCl with 1% BSA. After staining, cells were washed and analysed by flow cytometry with FACSVerse (BD Bioscience). Data were analysed using FlowJo software (Version 8.7.3).

### Rabbit erythrocyte-pVCC model

Erythrocytes were separated from whole rabbit blood (Preclinics) through ultracentrifugation, washed three times with PBS and kept in NaCl medium containing marimastat and AEBSF. For hemolysis assays and immunoblot analysis, 5% erythrocyte solution were prepared and incubated with pVCC (3 μg/ml) for 30 min on ice. Subsequently, erythrocytes were washed once with NaCl medium and split into the different samples. Aliquots received the individual ADAM-modulating agents (PPADS ‘before’ as shown in Figure 2C and D; OPS/OPC/TAPI/GI as shown in Figure 2E and F) at the given final concentrations for 10 min on ice. Afterwards, cells were stimulated with 0.5 mM BzATP for 15 min in the presence of marimastat to allow PS exposure but prevent shedding. Cells were washed and post-incubated at 37°C for 40 min in the presence of the indicated inhibitors (PPADS ‘after’ as shown in Figure 2C and D; OPS/OPC/TAPI/GI as shown in Figure 2E and F) in NaCl medium without marimastat. For hemolysis experiments, the cells were centrifuged at the indicated time periods and 100 μl supernatant was removed from each sample for analysis. Hemolysis was read at 405 nm (TECAN Sunrise). Western blot samples were probed as described previously. Samples were washed in 0.1× PBS and prepared for Gel-electrophoresis. pVCC cleavage was also quantified as percentage of ‘total’ VCC (mVCC plus pVCC) by densitometric analysis using PCBAS Software.

Flow cytometry analysis: Erythrocytes (1% solution) were stimulated with 0.5 mM BzATP for 15 min. Cells were transferred to ABB and stained with Annexin V-FITC (Enzo Life Sciences) for 10 min at RT. Erythrocytes were washed once with ABB and fixed with 1% PFA for 10 min at RT. Cells were again washed and transferred to NaCl medium for analysis with FACSVerse.

### Preparation of lipid aggregates/liposomes

Liposomes were prepared as 1 mg/ml (SAW, FRET) or 1 mM (ITC) aqueous dispersions of the phospholipids as follows. Lipids were dissolved in chloroform to a concentration of 10 mg/ml. After the solvent was evaporated under a stream of nitrogen and buffer was added, sonication with a Heinemann tip sonicator (G. Heinemann Ultraschall- und Labortechnik, Germany) for 4 min/ml (1 ml solution) was used to achieve homogeneous liposome formation. Subsequently, the preparation was cooled for 30 min at 4°C and two times heated for 30 min at 60°C and cooled to 4°C. Preparations were stored at 4°C overnight prior to measurements.

### Acoustic wave biochip analyses

Gold-coated chips (S-sens K5 Biosensor Quartz Chips, SAW Instruments GmbH, Germany) were functionalized as described (Andra et al., 2008). Biomolecular interaction processes on the surface of the sensor chip can affect phase and amplitude of the surface guided acoustic wave. Changes of these parameters correlate with mass loading and viscosity changes on the chip surface. Measurements were performed as described previously (Sommer et al., 2016b). To ensure a complete vesicle spreading process on the surface functionalization, the liposomes had to be prepared and dissolved in 5 mM HEPES buffer containing 150 mM NaCl. However, after membrane formation the buffer was displaced by 5 mM HEPES, pH 7.4. Since capturing of the liposomes is induced by electrostatic attraction between the positively charged PLL coupled to the sensor surface and negatively charged lipids the immobilization of pure PC liposomes was not possible in this setup. Therefore, a low amount of PS was added to PC. Following the immobilization of liposomes (300 μg/ml PS liposomes or PC:PS [9:1, M:M] liposomes) on the positively ionized sensor chip surface, 100 μl of 100 μg/ml solution of the ADAM10 peptide or a control peptide (KT peptide) with scrambled sequence in 5 mM HEPES were injected. Changes of phase and amplitude induced by the interaction of ADAM10 or the control peptide with the lipid bilayer were recorded over time. All biosensor measurements were performed at 22°C.

### FRET spectroscopy

Intercalation of the ADAM10 peptide or a control peptide (KT peptide) with scrambled sequence into liposomes was determined by FRET spectroscopy applied as a probe dilution assay (Gutsmann et al., 2000) using a Fluorolog 3 fluorescence spectrometer (HORIBA Jobin Yvon, USA). Liposomes were labelled by addition of 0.5% NBD-phosphatidylethanolamine (NBD-PE) as donor dye and 0.5% Rhodamine-DHPE as acceptor dye (molar ratio) to the chloroform lipid solution. The peptide was titrated 10 times in 10 μl injections of 1 mg/ml to 900 μl 10 μg/ml liposome dispersions (PS or PC:PS [9:1] in 5 mM HEPES, pH 7.4) at 37°C — leading to a 3:2 lipid:protein molar ratio after the last injection. By excitation of the system at 470 nm, the intercalation could be monitored as increase of the ratio between donor intensity I_d_ at 531 nm and acceptor intensity I_a_ at 593 nm (FRET signal) in a time-dependent manner.

### ITC

The interaction of the ADAM10 peptide or a control peptide (KT peptide) with scrambled sequence with PC or PS liposomes was analysed by ITC measurements on an ITC200 (GE Healthcare) (Brandenburg et al., 2005). Briefly, 0.5 mM solutions of the ADAM10 peptide in 5 mM HEPES were titrated 20 times in 2 μl injections to 250 μl 0.1 mM PS or PC liposome dispersions — leading to a 4:3 lipid:protein molar ratio after the last injection. The heat of dilution was determined in control experiments by injecting peptide solution into buffer (5 mM HEPES, pH 7.4). Enthalpy changes were recorded over time; measurements were performed at 37°C.

### Statistical analysis

All values for the ectodomain shedding assays are expressed as mean ± SEM. The SE values indicate the variation between mean values obtained from at least three independent experiments. Statistics were generated using two-tailed students *t*-test to compare control to treatment. Significant differences among groups were analysed using one-way ANOVA and Bonferroni multiple comparison *post hoc* test. *P*-values <0.05 were considered statistically significant (either indicated with * or #).

## Supplementary Material

mjz008_Supplementary_material
