# Supplementary material for: ADAM10 sheddase activation is controlled by cell membrane asymmetry
Source: J Mol Cell Biol. 2019 Feb 12;11(11):979–93. doi: 10.1093/jmcb/mjz008 (PMC6927242; doi:10.1093/jmcb/mjz008)
Supplement: mjz008_Supplementary_material [file mjz008_supplementary_material.pdf]

Supplementary Figure 1

A

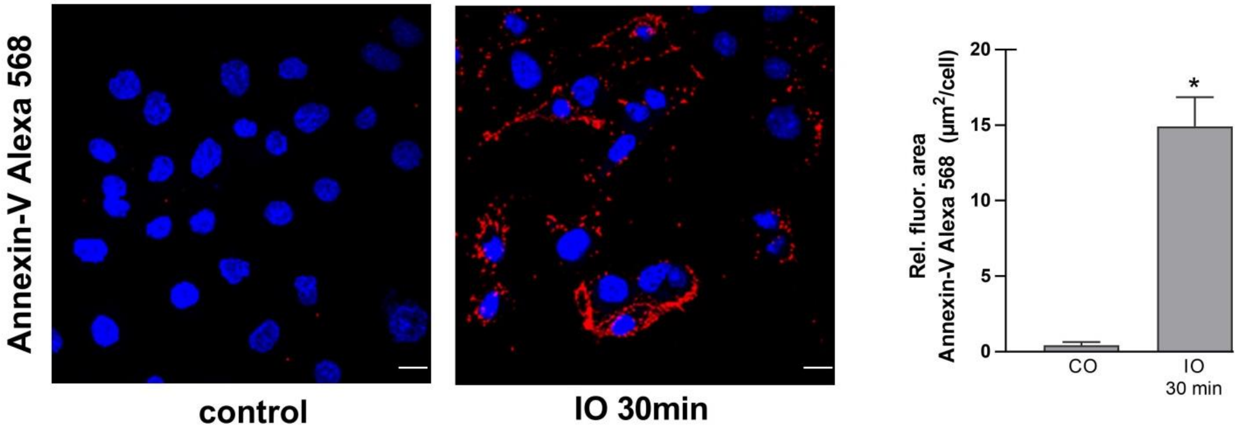

B

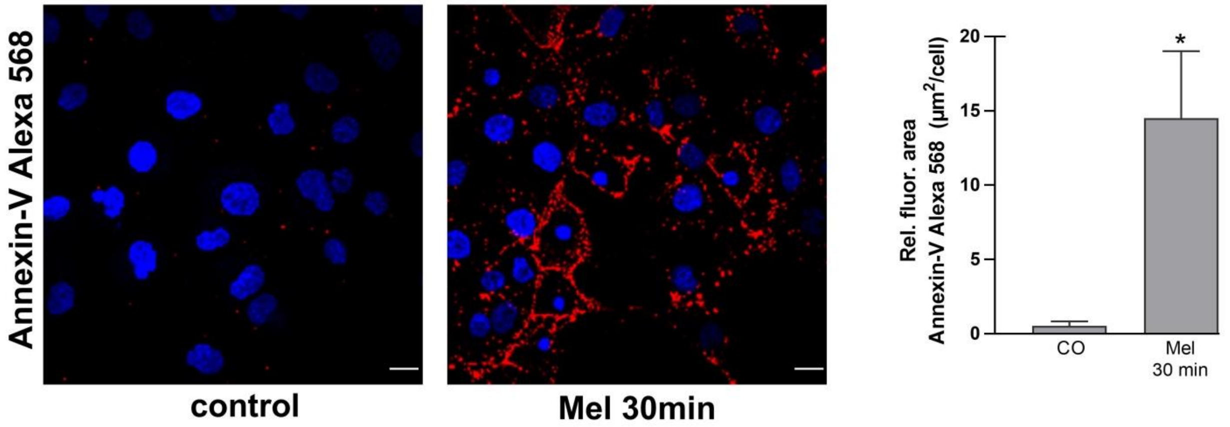

Supplementary Figure 1. Ionomycin and melittin induce PS exposure in COS7 cells. Cells were incubated for 30 minutes with (A) ionomycin (1 µM) or (B) melittin (0.5 µM) and analyzed for PS exposure using Annexin-V Alexa 568 staining. The mean fluorescence area was quantified for statistical analysis. Ionomycin and melittin led to significantly enhanced Annexin-V Alexa 568 signals upon stimulation (*$P<0.05$; $n=3$; ±s.e.m.). CO=control. Scale bars: 10 µm.

Supplementary Figure 2

A

B

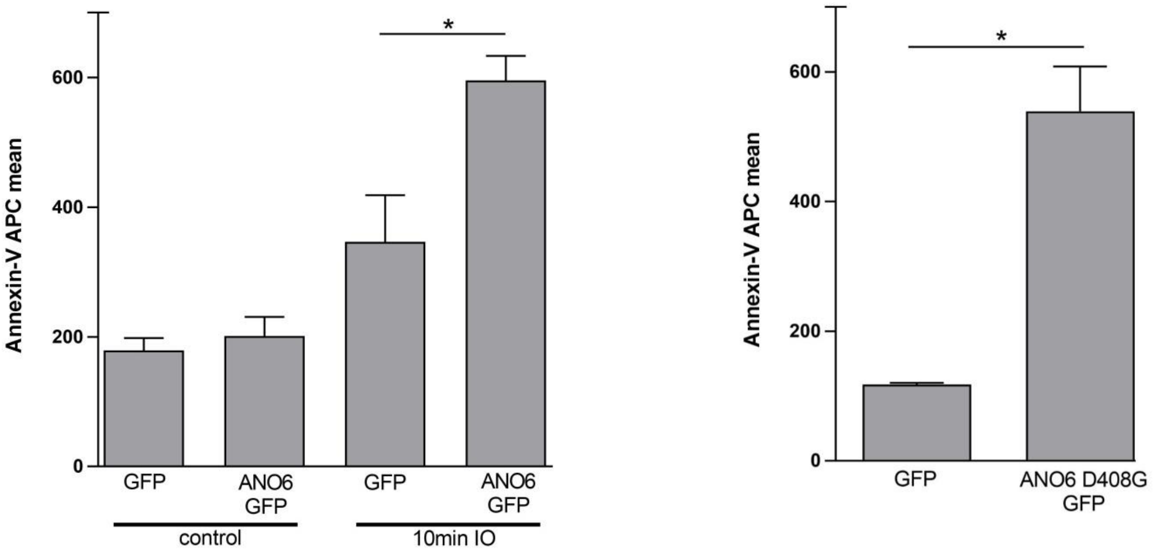

Supplementary Figure 2. Quantification of Annexin-V APC signals of ANO6 and ANO6-D408G-GFP overexpressing COS7 cells.

(A) COS7 cell were transfected with ANO6-GFP or GFP. Cells were stimulated with ionomycin (IO, 1 µM) for 10 minutes and labelled with Annexin-V APC. GFP-positive cells were analyzed via FACS for their Annexin-V APC signal. Quantification revealed significantly elevated PS levels of ANO6-GFP overexpressing cells compared to mock-transfected cells upon ionomycin treatment.

(B) COS7 cells were transfected with ANO6-D408G-GFP or GFP. Cells were labelled with Annexin-V APC. COS7 cells overexpressing ANO6-D408G-GFP showed significantly elevated Annexin-V APC signals in comparison to GFP overexpressing cells. All samples were analyzed via flow cytometry. Only GFP positive cells were analyzed for their Annexin-APC signal (*P<0.05; n=3; ±s.e.m.).

Supplementary Figure 3

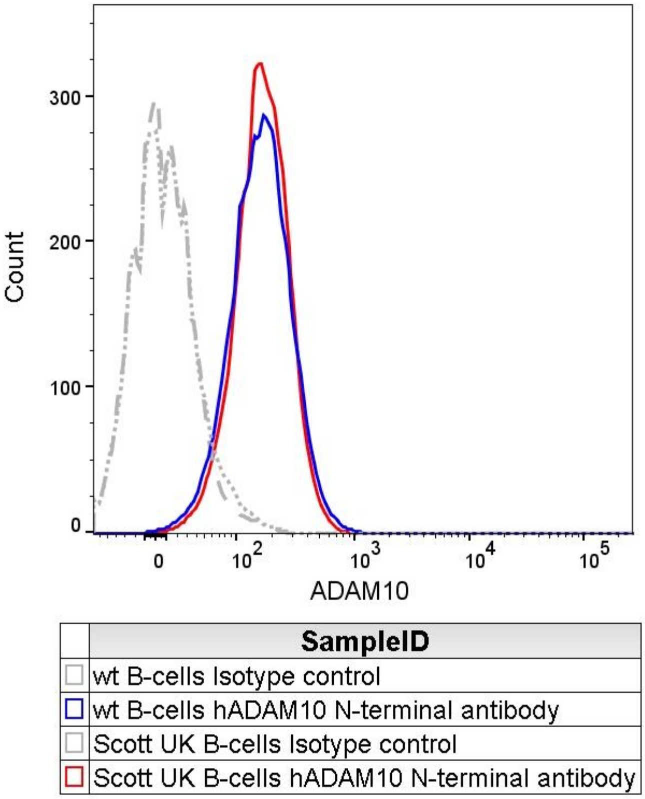

Supplementary Figure 3. Scott patient-cells and wild-type (wt) B-cell express comparable amounts of ADAM10. Scott UK B-cells and wild-type B-cells were labeled with hADAM10 N-terminal antibody or respective isotype control antibody and secondary antibody with fluorophore Alexa 488. Scott UK B-cells and wild-type B-cells express comparable levels of ADAM10 at the cell surface. All samples were analyzed via flow cytometry. One representative experiment out of three is shown.

Supplementary Figure 4

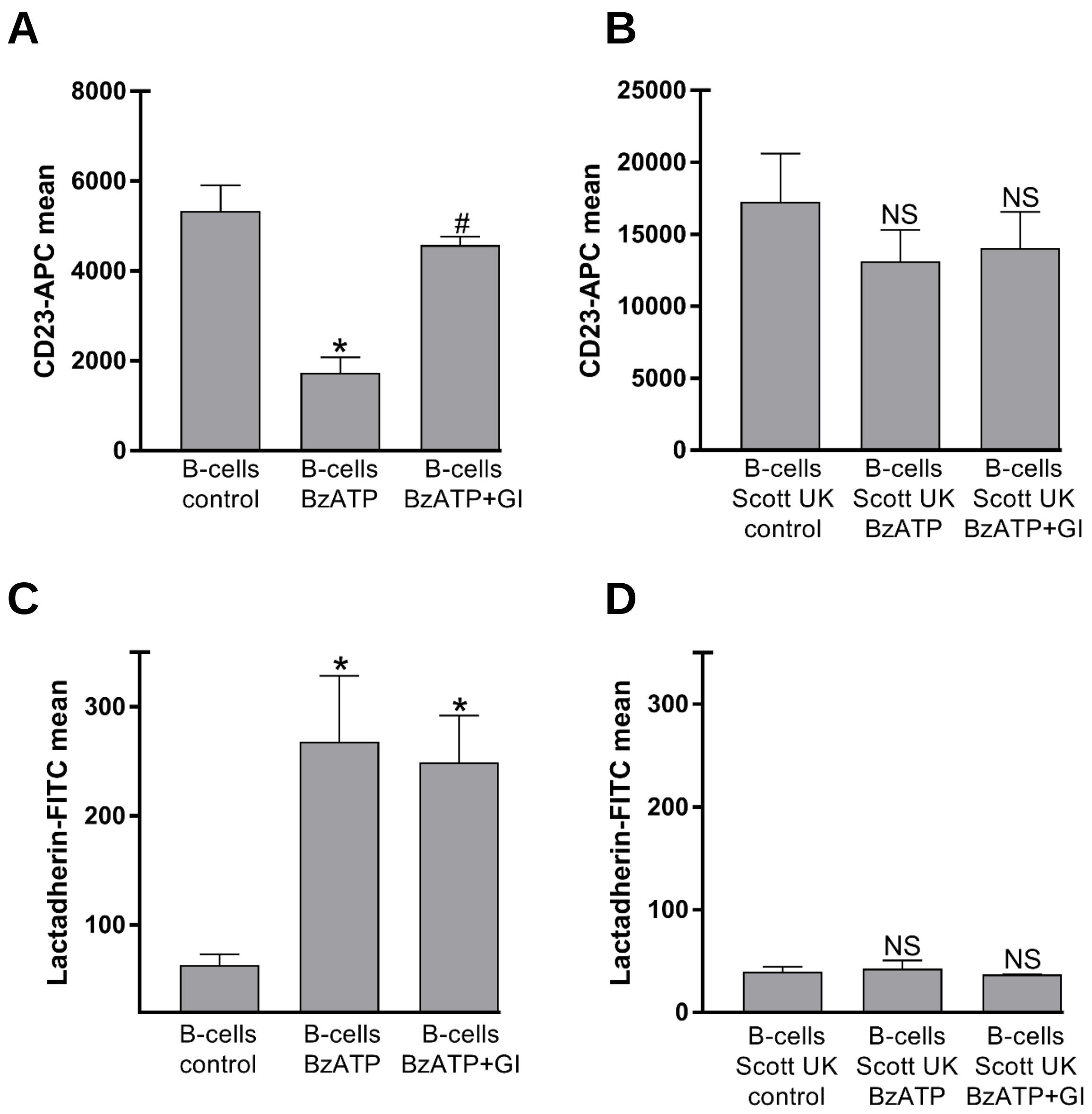

Supplementary Figure 4. Quantification of PS exposure (Lactaderin-FITC staining) and ADAM10-mediated shedding of CD23 from wild-type B-cells and Scott patient B-cells. Wild-type B-cells (A and C) and Scott patient B-cells (B and D) were stimulated with BzATP for 30 min and labeled with CD23-APC antibody (A and B) and Lactadherin-FITC (C and D). All samples were analyzed via flow cytometry. (A and B) * indicates significant decrease compared to control, # indicates significant increase compared to BzATP stimulation. (C and D) * indicates significant increase compared to control. NS=not significant. ($P<0.05$; $n=3$; ±s.e.m.).

Supplementary Figure 5

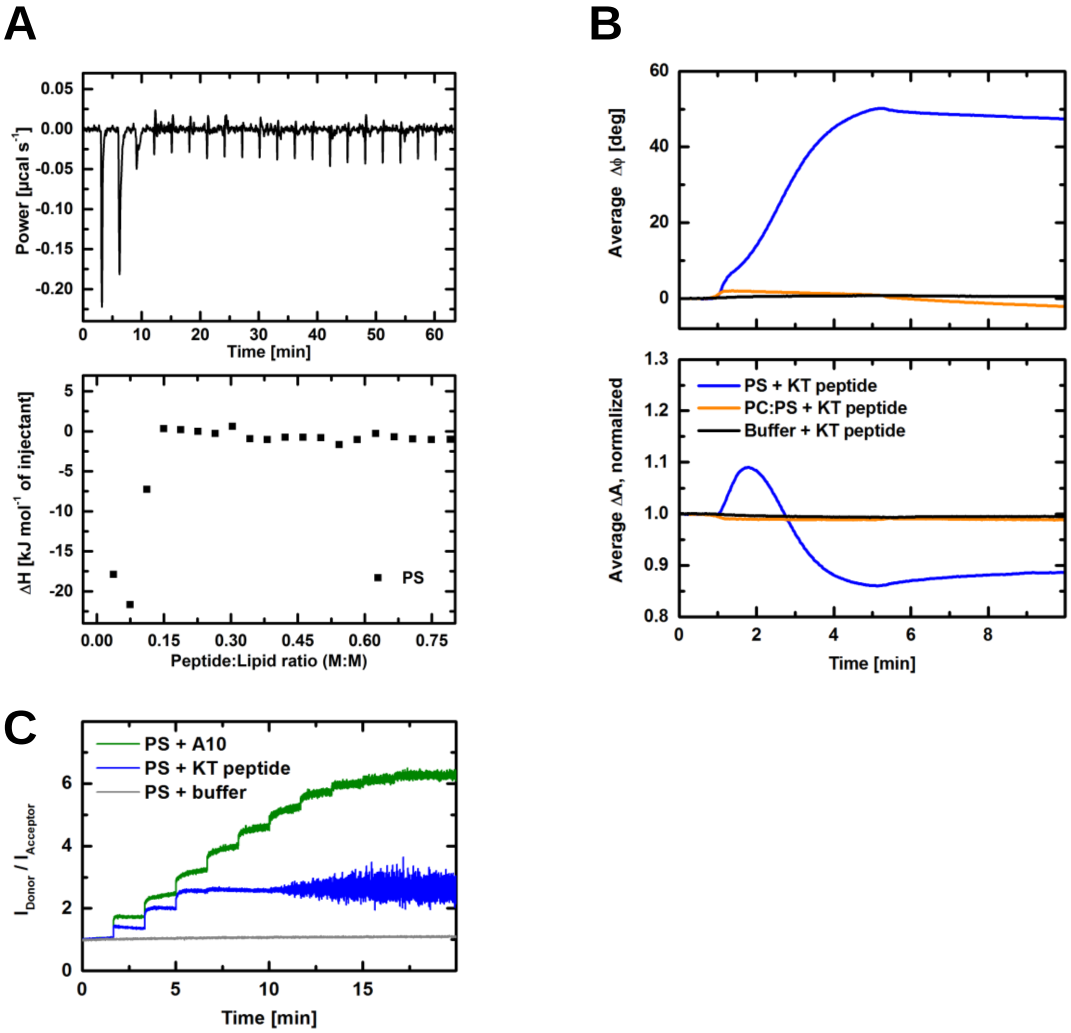

Supplementary Figure 5. Interaction of ADAM10 scrambled stalk region control peptide (KT peptide) with PS.

(A) ADAM10 scrambled stalk region control peptide (KT peptide) was incubated with PS-liposomes. ITC measurement showing the power resulting of the control peptide titration to PS liposomes as a function of time. The curve is representative for three independent measurements. The titration of the control peptide leads to an exothermic reaction, which becomes saturated at low peptide to lipid ratio. An endothermic reaction does not occur. (B) Injections of the ADAM10 control peptide on PS membranes (blue) or PC:PS (9:1) membranes (orange) or on the functionalization of the sensor chip (black) for SAW measurements. In comparison to the ADAM10 peptide, the control peptide binds more strongly to PS membranes and almost no binding to PC:PS membranes is visible (upper panel). The amplitude signal decreases after a first increase, which means that the viscosity of the system decreases (lower panel). The curves shown were averaged over five individual sensor channels and are representative of three independent experiments. (C) Data sets from FRET spectroscopy experiments showing the insertion of ADAM10 peptide (green) and control peptide (blue) into PS liposomes. The time course of the donor/acceptor intensity is shown as a measure for the insertion of peptides between the lipid molecules. While for ADAM10 peptide a clear intercalation occurs over ten injections up to a final concentration of 50 µg/ml, the control peptide leads already after the fourth injection to a stagnation of the intercalation, followed by an optically detectable precipitation of the reactants. This becomes visible by the significant increase in noise at t=10 min. The concentration of the vesicles was 10 µg/ml. The data shown are representative for three independent experiments.

Supplementary Figure 6

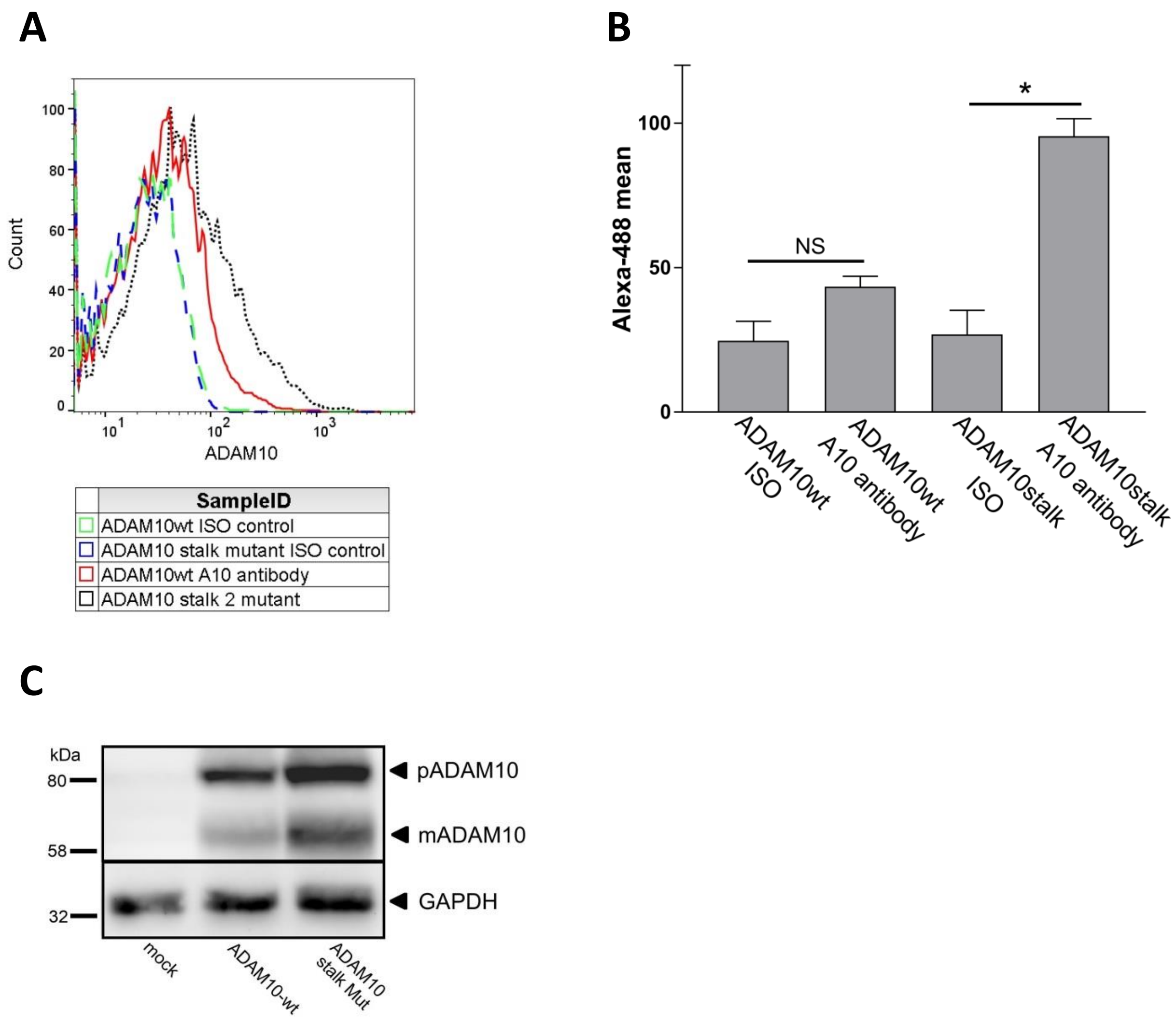

Supplementary Figure 6. Flow cytometry and Western Blot analysis of the ADAM10 stalk mutant. ADAM10 and ADAM17-deficient HEK-293T cells were transfected with ADAM10-wt or ADAM10 stalk mutant. (A) Transfected cells were labelled with ADAM10 N-terminal antibody or isotype control antibody and secondary antibody with fluorophore Alexa 488 and analyzed via flow cytometry. One representative out of three independent experiments is shown. (B) Quantification of ADAM10-wt and ADAM10 stalk Mut flow cytometry analysis of three independent experiments. (C) Western Blot analysis indicates expression of ADAM10-wt and ADAM10 stalk mutant pro (pADAM10) and mature form (mADAM10). GAPDH staining was used as loading control. One representative blot out of three independent experiments is shown. (*$P<0.05$; $n=3$; ±s.e.m.). NS=not significant.
